# Homogeneity Test for Correlated Binary Data

**DOI:** 10.1371/journal.pone.0124337

**Published:** 2015-04-21

**Authors:** Changxing Ma, Guogen Shan, Song Liu

**Affiliations:** 1 Department of Biostatistics, University at Buffalo, Buffalo, New York 14214, USA; 2 Department of Environmental and Occupational Health, School of Community Health Sciences, University of Nevada Las Vegas, Las Vegas, NV 89154, USA; 3 Department of Biostatistics and Bioinformatics, Roswell Park Cancer Institute, Buffalo, NY 14203, USA; The State University of New York at Stony Brook, UNITED STATES

## Abstract

In ophthalmologic studies, measurements obtained from both eyes of an individual are often highly correlated. Ignoring the correlation could lead to incorrect inferences. An asymptotic method was proposed by Tang and others (2008) for testing equality of proportions between two groups under Rosner's model. In this article, we investigate three testing procedures for general *g* ≥ 2 groups. Our simulation results show the score testing procedure usually produces satisfactory type I error control and has reasonable power. The three test procedures get closer when sample size becomes larger. Examples from ophthalmologic studies are used to illustrate our proposed methods.

## Introduction

In randomized clinical trials [2], patients are usually randomized into two or more treatment groups, and patients within each group receive the same treatment. Often a control group or a group with standard treatment is included for testing the efficiency of new treatments. After the randomization, all patients are followed up in exactly the same way as designed, and the only difference is the treatment assigned to each group. A randomized clinical trial is a good choice to eliminate many of the biases and to avoid ethical problems that may arise from comparing treatments [3] [4]. For example, in a double-blinded two-arm clinical trial for an ophthalmologic study, all patients are randomized into two treatment groups and the same treatment is applied to both eyes of patients from the same group. Such clustered data with a cluster size of two often arises from statistical and medical applications, for example, ophthalmologic studies, orthopaedic studies, otolaryngological studies and twin studies.

We wish to test if the outcomes are identical among the two or more treatment groups. Obviously, the information collected from two eyes of a single person tends to be highly correlated. Any statistical methods that ignore the feature of dependence, such as t tests, analyses of variance, or chi-square tests, could lead to incorrect inferences (see, [5] [6] [7] [8] [9]).

In this article, we consider the case of a dichotomous outcome, such as the presence of a disease or some other binary trait. Several statistical tests have been proposed. Rosner [5] proposed a parametric model and a test statistic for testing homogeneity of proportions among *g* groups. However, the maximum likelihood estimates (MLEs) and likelihood-based tests were not given. [1] [10] considered this problem for two groups only and proposed several asymptotic testing procedures, including the score test. It is difficult to extend the testing procedures from 2 groups to *g* groups (*g* > 2), due to the complexity of deriving the information matrix and maximum likelihood estimates which can be obtained only by numerical iterations. The score test statistic has demonstrated better type I error control and power than other testing procedures, when comparing two treatment groups [1]. We expect the score test, investigated for comparing multiple treatment groups in this article, to perform well as compared to other procedures.

In this article, we present the methods for comparing proportions among any *g* groups, with *g* ≥ 2. The maximum likelihood estimate under Rosner’s model and three different methods (Likelihood Ratio test, Wald-type test, Score test) are derived and investigated in Section 2. In Section 3, Monte Carlo simulation studies are conducted to compare the performance of various tests and comparisons are evaluated with respect to empirical type I error rates and powers. Examples from otolaryngological studies are illustrated to demonstrate our methodologies in Section 4. Finally, we give some concluding remarks in Section 5.

## Methods

Suppose we wish to compare *g* groups of individuals from an ophthalmologic study with *m*_*i*_ individuals in the *i*th group, *i* = 1, …, *g*; *N* = ∑*m*_*i*_ total subjects (Table 1). Let *Z*_*ijk*_ = 1 if the *k*th eye of *j*th individual in the *i*th group has a response at the end of the study, and 0 otherwise, *i* = 1, …, *g*, *j* = 1, …, *m*_*i*_, *k* = 1, 2. Let *m*_*li*_ denote the number of subjects who has exactly *l* responses in the *i*th group, and *S*_*l*_ be the number of subjects who has exactly *l* responses (e.g., affected eyes)

$$\begin{array}{c} S_{l}=\sum_{i=1}^{g}m_{li},l=0,1,2. \end{array}$$

**Table 1:** Frequencies of the number of affected eyes for persons in *g* groups.

	group				
number of affected eyes	1	2	⋯	g	total
0	*m*_01_	*m*_02_	⋯	*m*_0*g*_	*S*_0_
1	*m*_11_	*m*_12_	⋯	*m*_1*g*_	*S*_1_
2	*m*_21_	*m*_22_	⋯	*m*_2*g*_	*S*_2_
total	*m*_1_	*m*_2_	⋯	*m*_*g*_	*N*

A parametric model proposed by [5] is given as

$$\begin{array}{c} Pr\left(Z_{ijk}=1\right)=\pi_{i},Pr\left(Z_{ijk}=1|Z_{ij,3-k}=1\right)=R\pi_{i}, \end{array}$$ (1)

*i* = 1, …, *g*, *j* = 0, …, *m*_*i*_, *k* = 1, 2 for some positive *R*. The constant *R* is a measure of dependence between two eyes of the same person. If *R* = 1, the two eyes from the same patient are completely independent. If *Rπ*_*i*_ = 1, the eyes of each patient in the *i*-th group are completely dependent. The *R* satisfies 0 < *R* ≤ 1/*a*, if *a* ≤ 1/2; (2 − 1/*a*)/*a* ≤ *R* ≤ 1/*a*, if *a* > 1/2; where *a* = max{*π*_*i*_, *i* = 1, …, *g*}. From the conditional probability in Eq (1), it is easy to show that the correlation between two eyes is

$$\rho_{i}=corr\left(Z_{ij1},Z_{ij2}\right)=\frac{\pi_{i}}{1-\pi_{i}}\left(R-1\right),\;i=1,\;\ldots ,g.$$ (2)

We wish to test whether the response rates of the *g* groups are identical. The hypotheses are given as

$$\begin{array}{c} H_{0}:\pi_{1}=\cdots =\pi_{g}=\pi \end{array}$$

against

$$\begin{array}{c} H_{1}:\mathrm{some}\;\mathrm{of}\;\mathrm{the}\;\pi_{i}\;\mathrm{are}\;\mathrm{unequal}. \end{array}$$

Based on the observed data $\tilde{M}=(m_{01},\cdots ,m_{0g},m_{11},\cdots ,m_{1g},m_{21},\cdots ,m_{2g})$, the corresponding log-likelihood can be expressed as

$$\begin{array}{ccc} l(\pi_{1},\ldots ,\pi_{g};R) & = & \sum_{i=1}^{g}[m_{0i}\log(R\;{\pi_{i}}^{2}-2\;\pi_{i}+1)+m_{1i}\log(2\pi_{i}\left(1-R\pi_{i}\right))+m_{2i}\log(R\;\pi_{i}^{2})]. \end{array}$$

Differentiating *l*(*π*_1_, …, *π*_*g*_; *R*) with respect to parameters *π*_1_, …, *π*_*g*_ and *R* yields

$$\begin{array}{c} \frac{\partial l}{\partial \pi_{i}}=\frac{2\;m_{2i}}{\pi_{i}}+\frac{(2\;R\;\pi_{i}-2)\;m_{0i}}{R\;{\pi_{i}}^{2}-2\;\pi_{i}+1}+\frac{(4\;R\;\pi_{i}-2)\;m_{1i}}{2\;\pi_{i}\;(R\;\pi_{i}-1)},i=1,\ldots ,g \end{array}$$ (3)

$$\begin{array}{c} \frac{\partial l}{\partial R}=\sum_{i=1}^{g}(\frac{m_{2i}}{R}+\frac{{\pi_{i}}^{2}\;m_{0i}}{R\;{\pi_{i}}^{2}-2\;\pi_{i}+1}+\frac{\pi_{i}\;m_{1i}}{R\;\pi_{i}-1}) \end{array}$$ (4)

Under the null hypothesis *H*_0_ : *π*_1_ = ⋯ = *π*_*g*_ = *π*, the maximum likelihood estimates of *π* and *R* satisfy

$$\begin{array}{c} \frac{\partial l}{\partial R}=0\;\text{and}\;\frac{\partial l}{\partial \pi }=\frac{2\;S_{2}}{\pi }+\frac{(2\;R\;\pi -2)\;S_{0}}{R\;\pi^{2}-2\;\pi +1}+\frac{(4\;R\;\pi -2)\;S_{1}}{2\;\pi \;(R\;\pi -1)}=0, \end{array}$$

A direct algebra calculation results in the MLEs of $\pi_{i}^{\prime }s$ and *R*

$$\begin{array}{c} {\hat{\pi }}_{H_{0}}=\frac{S_{1}+2S_{2}}{2N} \end{array}$$

and

$$\begin{array}{c} {\hat{R}}_{H_{0}}=\frac{4NS_{2}}{{\left(S_{1}+2S_{2}\right)}^{2}}. \end{array}$$

Denote ${\hat{\pi }}_{i},i=1,\ldots ,g$ and $\hat{R}$ as the maximum likelihood estimate of *π*_*i*_, *i* = 1, …, *g* and *R*, respectively. ${\hat{\pi }}_{i},i=1,\ldots ,g$ and $\hat{R}$ are the solution of the following equations

$$\begin{array}{c} \frac{\partial l}{\partial \pi_{i}}=0,i=1,\ldots ,g,\;\;\;\;\;\frac{\partial l}{\partial R}=0. \end{array}$$

There is no closed form solution and it has to be solved iteratively. We can simplify the formula in Eq (3) as the following 3rd order polynomial (for *i* = 1, …, *g*)

$$\begin{array}{c} \pi_{i}^{3}-\frac{4m_{0i}+5m_{1i}+6m_{2i}}{2Rm_{i}}\pi_{i}^{2}+\frac{m_{0i}+\left(1+R\right)m_{1i}+\left(2+R\right)m_{2i}}{R^{2}m_{i}}\pi_{i}-\frac{m_{1i}+2m_{2i}}{2R^{2}m_{i}}=0 \end{array}$$

The (*t* + 1)th update for *π*_*i*_ can be directly obtained by the real root of the above equation, and *R* can be updated by the Fisher scoring method

$$\begin{array}{c} R^{\left(t+1\right)}=R^{\left(t\right)}-{\left(\frac{\partial^{2}l}{\partial R^{2}}\left(\pi_{1}^{\left(t\right)},\ldots ,\pi_{g}^{\left(t\right)};R^{\left(t\right)}\right)\right)}^{-1}\frac{\partial l}{\partial R}\left(\pi_{1}^{\left(t\right)},\ldots ,\pi_{g}^{\left(t\right)};R^{\left(t\right)}\right). \end{array}$$

See next section for the formula of $\frac{\partial^{2}l}{\partial R^{2}}$.

### Information matrix

Differentiating $\frac{\partial l}{\partial \pi_{i}},i=1,\ldots ,g$ and $\frac{\partial l}{\partial R}$ with respect to *π*_*i*_, *i* = 1, …, *g* and *R* respectively yields

$$\begin{array}{ccc} \frac{\partial^{2}l}{\partial \pi_{i}^{2}} & = & \frac{m_{0i}\;(-2\;R^{2}\;\pi_{i}^{2}+4\;R\;\pi_{i}+2\;R-4)}{{\left(R\;\pi_{i}^{2}-2\;\pi_{i}+1\right)}^{2}}-\frac{2\;m_{2i}}{\pi_{i}^{2}}-\frac{(2\;R^{2}\;\pi_{i}^{2}-2\;R\;\pi_{i}+1)\;m_{1i}}{\pi_{i}^{2}\;{\left(R\;\pi_{i}-1\right)}^{2}},\\ \frac{\partial^{2}l}{\partial \pi_{i}\partial R} & = & -\frac{m_{1i}}{{\left(R\;\pi_{i}-1\right)}^{2}}-\frac{2\;(\pi_{i}-1)\;\pi_{i}m_{0i}}{{\left(R\;{\pi_{i}}^{2}-2\;\pi_{i}+1\right)}^{2}},\\ & & \;\;\;\;\;\;i=1,\ldots ,g\\ \frac{\partial^{2}l}{\partial \pi_{i}\partial \pi_{j}} & = & 0,i\neq j,\\ \frac{\partial^{2}l}{\partial R^{2}} & = & -\frac{S_{2}}{R^{2}}-\sum_{i=1}^{g}\frac{\pi_{i}^{2}\;m_{1i}}{{\left(R\;\pi_{i}-1\right)}^{2}}-\sum_{i=1}^{g}\frac{\pi_{i}^{4}\;m_{0i}}{{\left(R\;\pi_{i}^{2}-2\;\pi_{i}+1\right)}^{2}}. \end{array}$$

Then we have

$$\begin{array}{ccc} I_{ii} & = & E(-\frac{\partial^{2}l}{\partial \pi_{i}^{2}})=\frac{2\;m_{i}\;(2\;R^{2}\;{\pi_{i}}^{2}-R\;{\pi_{i}}^{2}-2\;R\;\pi_{i}+1)}{\pi_{i}\;(R\;{\pi_{i}}^{2}-2\;\pi_{i}+1)(1-R\;\pi_{i})},\\ I_{i,g+1} & = & E(-\frac{\partial^{2}l}{\partial \pi_{i}\partial R})=-\frac{2\;(1-R)\;{\pi_{i}}^{2}\;m_{i}}{\left(R\;{\pi_{i}}^{2}-2\;\pi_{i}+1)(1-R\;\pi_{i}\right)},\\ & & \;\;\;\;\;\;i=1,\ldots ,g\\ I_{ij} & = & E(-\frac{\partial^{2}l}{\partial \pi_{i}\partial \pi_{j}})=0,i\neq j,\\ I_{g+1,g+1} & = & E(-\frac{\partial^{2}l}{\partial R^{2}})=\sum_{i=1}^{g}\frac{{\pi_{i}}^{2}\;m_{i}\left(R\pi_{i}-2\;\pi_{i}+1\right)}{R(R\;{\pi_{i}}^{2}-2\;\pi_{i}+1)(1-R\;\pi_{i})}. \end{array}$$

The (*g* + 1) × (*g* + 1) information matrix is denoted as *I*(*π*_1_, …, *π*_*g*_; *R*) = (*I*_*ij*_).

Under the null hypothesis *H*_0_ : *π*_1_ = ⋯ = *π*_*g*_ = *π*, it is straightforward but tedious to show that the inverse of the information matrix can be expressed as

$$\begin{array}{c} I^{-1}\left(\pi ;R\right)=\frac{\pi^{4}\;R\;{\left(R-1\right)}^{2}}{N(2\;\pi^{2}\;R^{2}-\pi^{2}\;R-2\;\pi \;R+1)}[\begin{array}{ccccc} c_{1} & 1 & \cdots & 1 & d\\ 1 & c_{2} & 1 & 1 & d\\ \cdots & \cdots & \cdots & \cdots & \cdots \\ 1 & 1 & \cdots & c_{g} & d\\ d & d & d & d & h \end{array}] \end{array}$$ (5)

where

$$\begin{array}{ccc} c_{i} & = & \frac{(R\;\pi^{2}-2\;\pi +1)\;(1-\pi \;R)N}{2\;\pi^{3}\;R\;{\left(R-1\right)}^{2}m_{i}}+1,i=1,\ldots ,g,\\ d & = & \frac{2\;\pi^{2}\;R^{2}-\pi^{2}\;R-2\;\pi \;R+1}{\pi^{3}\;(R-1)},\\ h & = & \frac{{\left(2\;\pi^{2}\;R^{2}-\pi^{2}\;R-2\;\pi \;R+1\right)}^{2}}{\pi^{6}\;{\left(R-1\right)}^{2}}. \end{array}$$

With the MLEs and information matrix derived, we consider the following test statistics.

### Likelihood ratio test (*T*_*LR*_)

The likelihood ratio (LR) test is given by

$$\begin{array}{c} T_{LR}=2\left[l\left({\hat{\pi }}_{1},\ldots ,{\hat{\pi }}_{g};\hat{R}\right)-l\left({\hat{\pi }}_{H_{0}},\ldots ,{\hat{\pi }}_{H_{0}};{\hat{R}}_{H_{0}}\right)\right]. \end{array}$$

Under the null hypothesis, *T*_*LR*_ is asymptotically distributed as a chi-square distribution with *g* − 1 degrees of freedom.

### Wald-type test (*T*_*W*_)

The null hypothesis *H*_0_ : *π*_1_ = ⋯ = *π*_*g*_ can be alternatively expressed as *C* ***β*** ^*T*^ = 0 where ***β*** = (*π*_1_, ⋯, *π*_*g*_, *R*) and

$$\begin{array}{c} C={\left[\begin{array}{cccccc} 1 & -1 & & & & 0\\ & 1 & -1 & & & 0\\ & & \ddots & \ddots & & \vdots \\ & & & 1 & -1 & 0 \end{array}\right]}_{g-1,g}. \end{array}$$

Wald-type test statistic (*T*_*W*_) for testing *H*_0_ can be expressed as

$$\begin{array}{c} T_{W}=\left(\beta C^{T}\right){\left(CI^{-1}C^{T}\right)}^{-1}\left(C\beta^{T}\right)|\beta =\left({\hat{\pi }}_{1},\ldots ,{\hat{\pi }}_{g},\hat{R}\right), \end{array}$$

where *I* is the Fisher information matrix for and *T*_*W*_ is asymptotically distributed as a chi-square distribution with *g* − 1 degrees of freedom. *T*_*W*_ can be simplified as

$$\begin{array}{c} T_{W}=\frac{\sum_{i,j=1}^{g}{\hat{\pi }}_{i}{\hat{\pi }}_{j}D_{ij}}{\sum_{k=1}^{g}\left(b_{k}^{2}-ha_{k}\right)}, \end{array}$$

where

$$\begin{array}{ccc} D_{ij} & = & \left\{ \begin{array}{cc} \left(b_{i}^{2}-ha_{i}\right)\sum_{k\neq i}a_{k}+a_{i}{\left(\sum_{k\neq i}b_{i}\right)}^{2}, & \text{if}\;i=j,\\ b_{i}a_{j}\sum_{k\neq j}b_{k}+b_{j}a_{i}\sum_{k\neq i}b_{k}-ha_{i}a_{j}-b_{i}b_{j}\sum_{k\neq i,j}a_{k}, & \text{if}\;i\neq j, \end{array}\right.\\ a_{k} & = & \frac{-2m_{k}\left(2{\hat{R}}^{2}{\hat{\pi }}_{k}^{2}-\hat{R}{\hat{\pi }}_{k}^{2}-2\hat{R}{\hat{\pi }}_{k}+1\right)}{{\hat{\pi }}_{k}\left({\hat{R}}^{2}{\hat{\pi }}_{k}^{3}-3\hat{R}{\hat{\pi }}_{k}^{2}+\hat{R}{\hat{\pi }}_{k}+2{\hat{\pi }}_{k}-1\right)},\\ b_{k} & = & \frac{2m_{k}\left(1-\hat{R}\right){\hat{\pi }}_{k}^{2}}{{\hat{R}}^{2}{\hat{\pi }}_{k}^{3}-3\hat{R}{\hat{\pi }}_{k}^{2}+\hat{R}{\hat{\pi }}_{k}+2{\hat{\pi }}_{k}-1},\\ h & = & \sum_{i=1}^{g}m_{i}[\frac{{\hat{\pi }}_{i}^{4}}{1-2{\hat{\pi }}_{i}+\hat{R}{\hat{\pi }}_{i}^{2}}+\frac{{\hat{\pi }}_{i}^{2}}{\hat{R}}+\frac{2{\hat{\pi }}_{i}^{3}}{1-\hat{R}{\hat{\pi }}_{i}}]. \end{array}$$

Other multivariate tests of *π*’s can be similarly done by choosing the corresponding *C* matrix in the above statistic. Further, a Wald-type test statistic for testing *H*_0*a*_ : *π*_*i*_ = *π*_*j*_ vs *H*_1*a*_ : *π*_*i*_ ≠ *π*_*j*_, *i* ≠ *j* can be given by

$$\begin{array}{c} T_{Wa}\left(i,j\right)=\left(\beta c^{T}\right){\left(cI^{-1}c^{T}\right)}^{-1}\left(c\beta^{T}\right)|\beta =\left({\hat{\pi }}_{1},\ldots ,{\hat{\pi }}_{g},\hat{R}\right), \end{array}$$

where *c* = (0, …, 1, …, −1, …, 0) with 1 in the *i*th element and −1 in the *j*th element. *T*_*Wa*_ is asymptotically distributed as a chi-square distribution with 1 degree of freedom. *T*_*Wa*_(*i*, *j*) can be simplified as

$$\begin{array}{c} T_{Wa}\left(i,j\right)=\frac{a_{i}a_{j}{\left({\hat{\pi }}_{i}-{\hat{\pi }}_{j}\right)}^{2}\left(\sum_{k=1}^{g}\left(b_{k}^{2}/a_{k}\right)-h\right)}{\left(a_{i}+a_{j}\right)\left(\sum_{k\neq i,j}^{g}\left(b_{k}^{2}/a_{k}\right)-h\right)+{\left(b_{i}+b_{j}\right)}^{2}}. \end{array}$$

### Score test (*T*_*SC*_)

The score test statistic *T*_*SC*_ is given by

$$\begin{array}{c} T_{SC}^{2}=UI{\left(\pi ,R\right)}^{-1}U^{T}|\pi_{1}=\cdots =\pi_{g}={\hat{\pi }}_{H_{0}},R={\hat{R}}_{H_{0}} \end{array}$$

where

$$\begin{array}{c} U=(\frac{\partial l}{\partial \pi_{1}},\ldots ,\frac{\partial l}{\partial \pi_{g}},0) \end{array}$$

and see (Eq 5) for the formula of the inverse of the information matrix *I*(*π*, *R*)^−1^.

It can be simplified as

$$\begin{array}{c} T_{SC}^{2}=\sum_{k=1}^{g}\frac{N{\left(S_{1}^{2}m_{0k}-S_{0}S_{1}\left(m_{1k}+2m_{2k}\right)+2S_{0}S_{2}m_{1k}\right)}^{2}}{S_{0}S_{1}(S_{1}^{3}+S_{0}S_{1}^{2}+4S_{0}S_{2}^{2})m_{k}} \end{array}$$ (6)

after lengthy algebra calculations. $T_{SC}^{2}$ is asymptotically distributed as a chi-square distribution with *g* − 1 degree of freedom.

*Remark* 1. One limitation of the score statistic is that it cannot be computed if *S*_0_ = 0 or *S*_1_ = 0. We dealt with this problem by adding 1/(2*g*) to *m*_*ij*_ for such situations.

*Remark* 2. [1] derived a score test *T*_*SC*_ for *g* = 2 as

$$\begin{array}{c} T_{SC}=\frac{N[S_{0}S_{1}\left(m_{11}+2m_{21}\right)-m_{01}S_{1}^{2}-2m_{11}S_{0}S_{2}]}{\sqrt{m_{1}m_{2}S_{0}S_{1}\left[S_{1}^{2}\left(S_{0}+S_{1}\right)+4S_{0}S_{2}^{2}\right]}}, \end{array}$$

which is equivalent to (Eq 6).

## Monte Carlo simulation studies

We now investigate the performance of proposed statistics and testing procedures discussed in the previous section. First, we investigate the behavior of the type I error rates of various procedures for *g* = 2,3,4,5; sample size *m*_1_ = ⋯ = *m*_*g*_ = 20, 40, 60, 80 and 100; *π*_1_ = ⋯ = *π*_*g*_ = *π*_0_ = 0.5(0.1)0.8; and *R* = 1 + *ρ*(1 − *π*_0_)/*π*_0_ where *ρ* = 0.4(0.1)0.6. An imbalanced sample setting is also considered. In each configuration, 50,000 replications are generated based on the null hypothesis, and empirical type I error rates are computed as *the number of rejections*/50000. The results are presented in Table 2. Following [1], we calculated the ratio of empirical type I error rate to the nominal type I error rate. A test is said to be liberal if the ratio is greater than 1.2 (i.e., > 6% for *α* = 5%), conservative if the ratio is less than 0.8 (i.e., < 4%), and robust if the ratio is between 0.8 and 1.2.

**Table 2:** The type I error rates (percent) of various procedures under *H*_0_ : *π*_1_ = ⋯ = *π*_*g*_ = *π*_0_ at *α* = 0.05 based on 50,000 replicates.

*m*	*π*_0_	*ρ*	*g* = 2			*g* = 3			*g* = 4			*g* = 5		
			$T_{LR}^{2}$	$T_{W}^{2}$	$T_{SC}^{2}$	$T_{LR}^{2}$	$T_{W}^{2}$	$T_{SC}^{2}$	$T_{LR}^{2}$	$T_{W}^{2}$	$T_{SC}^{2}$	$T_{LR}^{2}$	$T_{W}^{2}$	$T_{SC}^{2}$
20	0.5	0.4	**6.70**	**6.63**	5.39	**6.95**	**8.09**	5.06	**7.10**	**9.64**	4.99	**7.19**	**10.66**	5.05
		0.5	**6.70**	5.76	5.34	**7.11**	**7.66**	5.10	**7.34**	**9.48**	5.01	**7.34**	**10.81**	4.82
		0.6	**7.11**	4.86	5.35	**7.27**	**6.94**	4.80	**7.72**	**9.51**	4.79	**8.19**	**11.97**	4.92
	0.6	0.4	**6.63**	**6.51**	5.25	**6.80**	**8.18**	5.16	**7.05**	**9.42**	5.15	**6.87**	**10.44**	4.80
		0.5	**6.64**	5.57	5.32	**6.82**	**7.14**	4.95	**6.96**	**8.93**	4.82	**7.38**	**10.45**	4.92
		0.6	**7.18**	4.58	5.37	**7.34**	**6.95**	4.91	**7.64**	**9.29**	4.89	**7.80**	**11.54**	4.85
	0.7	0.4	**6.46**	**6.28**	4.76	**6.79**	**7.96**	5.05	**6.85**	**9.28**	4.92	**6.99**	**10.55**	4.77
		0.5	**6.90**	5.58	5.11	**6.89**	**7.23**	4.90	**7.30**	**9.06**	4.77	**7.64**	**10.98**	4.91
		0.6	**7.46**	4.43	5.02	**7.72**	**6.94**	4.80	**7.97**	**10.36**	4.73	**8.42**	**13.80**	4.92
	0.8	0.4	**6.76**	**6.51**	4.94	**7.26**	**8.35**	4.85	**7.49**	**10.37**	4.81	**7.71**	**12.15**	4.75
		0.5	**7.58**	5.45	4.99	**7.92**	**7.89**	4.78	**8.04**	**11.50**	4.72	**8.24**	**14.92**	4.65
		0.6	**7.81**	4.00	4.37	**8.31**	**6.94**	4.46	**8.15**	**11.87**	4.42	**8.28**	**17.56**	4.52
40	0.5	0.4	5.72	5.61	5.07	6.00	**6.42**	5.16	5.86	**7.01**	4.97	6.00	**7.66**	5.03
		0.5	5.71	5.11	5.15	5.84	5.89	4.98	5.90	**6.80**	4.91	**6.11**	**7.55**	4.92
		0.6	5.76	4.66	5.13	5.84	5.48	4.98	**6.12**	**6.62**	4.97	**6.20**	**7.45**	5.02
	0.6	0.4	5.58	5.48	4.98	5.72	**6.08**	5.05	5.83	**6.90**	4.98	5.87	**7.48**	4.99
		0.5	5.74	5.13	5.14	5.82	5.83	5.03	**6.04**	**6.73**	5.18	**6.16**	**7.57**	5.13
		0.6	5.84	4.73	5.19	5.79	5.37	5.00	5.98	**6.49**	4.94	**6.05**	**7.43**	4.97
	0.7	0.4	5.62	5.41	5.03	5.84	**6.11**	5.13	5.58	**6.42**	4.85	5.71	**7.30**	4.89
		0.5	5.59	4.94	4.96	5.88	5.79	5.21	5.86	**6.65**	5.05	5.69	**7.28**	4.89
		0.6	5.74	4.56	5.00	5.73	5.60	4.88	**6.01**	**6.76**	5.00	**6.08**	**7.59**	4.97
	0.8	0.4	5.76	5.33	5.30	5.85	**6.16**	4.99	5.98	**7.19**	5.10	6.00	**8.00**	5.04
		0.5	5.58	4.63	4.97	5.68	5.78	4.76	5.81	**6.87**	4.68	**6.00**	**7.79**	4.92
		0.6	5.85	4.44	4.91	5.91	5.71	4.67	**6.20**	**7.23**	4.77	**6.43**	**8.51**	4.71
60	0.5	0.4	5.39	5.20	4.94	5.67	**6.02**	5.06	5.60	**6.27**	4.95	5.62	**6.77**	4.91
		0.5	5.57	5.10	5.11	5.85	5.83	5.23	5.75	**6.28**	5.02	5.74	**6.59**	4.99
		0.6	5.58	4.67	5.06	5.60	5.28	5.02	5.84	**6.08**	5.15	5.51	**6.39**	4.79
	0.6	0.4	5.57	5.46	5.19	5.57	5.77	5.06	5.38	**6.09**	4.86	5.80	**6.75**	5.14
		0.5	5.36	4.90	4.97	5.42	5.39	5.01	5.55	5.85	4.94	5.57	**6.39**	4.91
		0.6	5.56	4.79	5.21	5.56	5.33	4.98	5.52	5.78	4.93	5.69	**6.62**	4.95
	0.7	0.4	5.46	5.24	5.09	5.51	5.62	5.10	5.60	**6.23**	5.08	5.44	**6.47**	4.85
		0.5	5.44	4.99	5.10	5.54	5.51	5.08	5.48	**6.02**	4.97	5.47	**6.55**	4.85
		0.6	5.42	4.70	5.04	5.32	5.14	4.84	5.50	5.82	4.91	5.47	**6.57**	4.82
	0.8	0.4	5.41	5.05	5.07	5.52	5.69	5.01	5.47	**6.19**	4.94	5.50	**6.76**	4.97
		0.5	5.42	4.79	5.04	5.67	5.58	5.19	5.61	**6.14**	4.98	5.53	**6.91**	4.80
		0.6	5.36	4.56	4.87	5.76	5.59	5.08	5.78	**6.54**	4.88	5.81	**7.33**	4.94
80	0.5	0.4	5.29	5.17	4.95	5.56	5.75	5.12	5.61	**6.21**	5.13	5.50	**6.32**	5.00
		0.5	5.25	4.88	4.91	5.34	5.37	5.02	5.43	5.75	4.90	5.53	**6.14**	4.96
		0.6	5.34	4.72	5.05	5.49	5.26	5.08	5.50	5.70	4.93	5.59	**6.09**	5.04
	0.6	0.4	5.46	5.34	5.23	5.41	5.51	4.99	5.25	5.66	4.82	5.38	**6.10**	4.98
		0.5	5.32	4.91	5.02	5.26	5.25	4.96	5.47	5.77	5.16	5.37	5.98	4.97
		0.6	5.31	4.75	4.98	5.35	5.12	5.00	5.59	5.81	5.15	5.35	**6.04**	4.92
	0.7	0.4	4.99	4.78	4.75	5.30	5.41	4.97	5.30	5.74	4.92	5.45	**6.24**	4.98
		0.5	5.35	4.93	5.13	5.36	5.26	5.06	5.58	5.96	5.16	5.37	6.00	4.90
		0.6	5.38	4.83	5.13	5.41	5.38	5.03	5.39	5.83	4.91	5.42	**6.01**	4.86
	0.8	0.4	5.32	4.97	5.08	5.47	5.51	5.09	5.35	5.81	4.97	5.40	**6.22**	4.96
		0.5	5.04	4.61	4.83	5.51	5.44	5.16	5.60	**6.05**	5.14	5.27	**6.22**	4.86
		0.6	5.19	4.64	4.92	5.39	5.40	4.86	5.46	6.00	4.90	5.49	**6.52**	4.86
100	0.5	0.4	5.23	5.09	4.94	5.27	5.38	4.98	5.34	5.81	4.98	5.21	5.90	4.72
		0.5	5.41	5.13	5.17	5.40	5.44	5.01	5.32	5.64	4.98	5.44	5.96	4.99
		0.6	5.39	4.85	5.16	5.45	5.25	5.14	5.28	5.38	4.83	5.47	5.78	4.95
	0.6	0.4	5.33	5.23	5.13	5.42	5.63	5.18	5.46	5.79	5.15	5.17	5.82	4.83
		0.5	5.11	4.85	4.91	5.40	5.29	5.02	5.39	5.62	5.07	5.13	5.66	4.79
		0.6	5.26	4.87	5.07	5.48	5.23	5.21	5.37	5.48	4.96	5.09	5.53	4.76
	0.7	0.4	5.37	5.16	5.18	5.15	5.23	4.85	5.44	5.74	5.05	5.35	5.87	5.08
		0.5	5.17	4.90	5.00	5.15	5.08	4.91	5.16	5.47	4.86	5.28	5.83	4.96
		0.6	5.50	5.06	5.29	5.38	5.27	5.08	5.36	5.61	4.99	5.30	5.87	4.93
	0.8	0.4	5.33	5.09	5.09	5.04	5.12	4.78	5.29	5.72	4.91	5.31	5.93	4.95
		0.5	5.16	4.79	4.93	5.23	5.21	4.97	5.45	5.84	5.03	5.38	**6.21**	4.88
		0.6	5.18	4.72	4.88	5.38	5.31	4.96	5.62	**6.04**	5.19	5.38	**6.35**	4.85
*	0.5	0.4	**6.32**	**6.87**	5.30	**6.14**	**7.42**	4.92	**6.62**	**8.69**	5.15	**6.29**	**9.33**	4.81
		0.5	**6.44**	**6.54**	5.29	**6.49**	**7.30**	5.05	**6.60**	**8.47**	4.93	**6.62**	**9.50**	4.86
		0.6	**6.54**	**6.07**	5.13	**6.58**	**7.11**	4.93	**6.88**	**8.66**	4.91	**6.88**	**9.76**	4.95
	0.6	0.4	5.91	**6.42**	5.07	**6.12**	**7.34**	4.98	**6.33**	**8.40**	5.04	**6.40**	**9.31**	5.10
		0.5	6.00	**6.16**	4.95	**6.48**	**7.39**	5.19	**6.61**	**8.31**	5.17	**6.32**	**9.10**	4.77
		0.6	**6.16**	5.99	4.85	**6.52**	**6.91**	4.97	**7.15**	**8.54**	5.06	**6.91**	**9.69**	4.92
	0.7	0.4	**6.00**	**6.44**	5.13	**6.17**	**7.36**	5.09	**6.19**	**8.30**	4.88	**6.26**	**9.26**	4.82
		0.5	**6.26**	**6.24**	5.19	**6.30**	**7.25**	4.91	**6.50**	**8.20**	4.83	**6.75**	**9.48**	5.11
		0.6	**6.56**	**6.15**	5.04	**6.75**	**7.28**	4.90	**6.99**	**8.90**	4.77	**6.58**	**9.90**	4.67
	0.8	0.4	**6.20**	**6.74**	5.01	**6.48**	**7.60**	5.07	**6.78**	**9.04**	4.92	**6.71**	**9.93**	4.81
		0.5	**6.77**	**6.71**	5.04	**6.69**	**7.65**	4.72	**7.09**	**9.59**	4.80	**6.72**	**10.54**	4.62
		0.6	**7.16**	**7.08**	4.63	**7.50**	**9.41**	4.83	**7.75**	**12.24**	4.78	**7.36**	**14.32**	4.60

* unequal sample sizes: (*m*_1_, …, *m*_*g*_) = (20,40), (20,30,40), (20, 25, 30, 35), (20,25,30,35,40) for *g* = 2,3,4,5, respectively.

Generally, score tests $T_{SC}^{2}$ produce satisfactory type I error controls for any configuration while LR tests and Wald tests are liberal, especially for small samples and larger numbers of groups (*g*). When *g* > 2, Wald tests are more liberal than LR tests and these tests get closer when sample size becomes larger.

LR tests and Wald-type tests are extremely liberal for a small sample size (i.e., *m* = 20), and their actual sizes inflate with the increase of the correlation coefficient (i.e., *ρ*).

Next, we evaluate the power performance of proposed methods. We consider the alternative hypotheses with *H*_1_ : *π* = (0.25, 0.4), (0.25, 0.325, 0,4), and (0.25, 0.3, 0.35, 0.4) for *g* = 2, 3, and 4, respectively. *R* is chosen as 1, 1.5, and 2.0 and sample size *m*_1_ = ⋯ = *m*_*g*_ = 20, 40, 60, 80 and 100. Ronser’s statistic *T* is also considered in the simulation studies and the results are presented in Table 3.

**Table 3:** The power (percent) of various procedures at *α* = 0.05 based on 50,000 replicates.

*m*	*R*	*g* = 2				*g* = 3				*g* = 4			
		$T_{SC}^{2}$	$T_{LR}^{2}$	$T_{W}^{2}$	*T*	$T_{SC}^{2}$	$T_{LR}^{2}$	$T_{W}^{2}$	*T*	$T_{SC}^{2}$	$T_{LR}^{2}$	$T_{W}^{2}$	*T*
20	1.0	29.5	31.7	33.5	30.2	22.1	24.8	28.7	22.7	20.6	23.5	28.5	20.9
	1.5	24.7	29.2	29.9	24.5	18.7	23.2	26.0	18.7	17.4	22.1	26.6	16.8
	2.0	30.0	36.4	29.8	21.3	23.6	30.8	27.4	16.1	21.9	29.4	29.4	14.8
40	1.0	52.2	53.5	54.9	52.8	41.9	43.6	46.1	42.3	39.9	41.7	44.7	40.1
	1.5	45.6	48.7	49.3	44.1	35.9	39.5	41.3	34.2	34.4	37.8	40.7	32.3
	2.0	55.9	60.0	55.6	37.6	45.8	50.6	48.3	29.1	43.7	48.7	48.2	27.2
60	1.0	69.8	70.6	71.5	70.2	59.4	60.6	62.3	60.0	57.5	58.8	60.9	57.8
	1.5	62.7	64.8	65.3	60.1	52.3	54.8	56.3	49.5	50.4	53.1	55.2	47.3
	2.0	74.3	76.6	74.4	52.6	64.8	68.2	66.8	42.0	63.4	66.9	66.2	40.2
80	1.0	81.7	82.2	82.7	82.1	72.9	73.7	74.7	73.2	71.4	72.2	73.6	71.5
	1.5	75.9	77.2	77.6	72.7	65.8	67.6	68.7	62.3	64.7	66.6	68.0	61.0
	2.0	86.1	87.2	86.1	64.5	78.4	80.6	79.7	53.8	78.0	80.2	79.9	52.3
100	1.0	89.4	89.6	89.9	89.6	82.6	83.1	83.7	82.7	82.0	82.5	83.4	82.1
	1.5	84.8	85.6	85.9	82.1	76.4	77.7	78.5	73.0	75.5	76.9	77.9	71.6
	2.0	92.8	93.4	92.8	74.3	87.4	88.9	88.4	64.6	87.4	88.8	88.6	63.0
*H*_1_ :	*π* =	(0.25, 0.4)				(0.25, 0.325, 0,4)				(0.25, 0.3, 0.35, 0.4)			

Note: *T* is the test statistic [5].

Based on the simulation results, LR and Wald tests are generally more powerful than score tests and Ronser’s *T* generally has less power. However, power of LR and Wald tests is often overestimated in small sample size due to the inflated type I error rates from these tests being observed (see Table 2). For moderate or large sample sizes, the powers of the three proposed methods are close. Overall, the score test is highly recommended as it has reasonable power with satisfactory type I error control.

## Examples

We first reanalyze the data presented by [5] to illustrate the newly proposed methods. The data consists of 218 persons aged 20–39 with retinitis pigmentosa (RP) who were seen at Massachusetts Eye and Ear Infirmary. They were classified into four genetic types, namely, autosomal dominant RP (DOM), autosomal recessive RP (AR), sex-linked RP (SL), and isolate RP (ISO). The differences between these four groups on the Snellen visual acuity (VA) were assessed. An eye was considered affected if VA was 20/50 or worse, and normal if VA was 20/40 or better. The sample used for this analysis consists of 216 persons out of the sample of 218 persons each of whom had complete information for VA on both eyes (Table 4).

**Table 4:** Distribution of the number of affected eyes for persons in each genetic type [5].

	genetic type			
number of affected eyes	DOM	AR	SL	ISO
0	15	7	3	67
1	6	5	2	24
2	7	9	14	57

An overall significant difference between the proportions of affected eyes in the four groups is from 0.0769 to 0.1173 based on proposed methods and 0.010 on Rosner’s statistic *T* (Table 5).

**Table 5:** Statistic and p-value for comparing VA for different genetic types of RP.

method	$T_{LR}^{2}$	$T_{W}^{2}$	$T_{SC}^{2}$	*T*
statistic	5.8862	6.2966	6.8475	11.36
p-value	0.1173	0.0980	0.0769	0.010

The maximum likelihood estimates and pairwise comparisons based on Wald-type test *T*_*Wa*_(*i*, *j*) are shown in Table 6. It shows a significant difference between DOM and AR (p = 0.0207).

**Table 6:** Wald-type test results comparing VA for different genetic types of RP.

Group *i*	*MLE* ${\hat{\pi }}_{i}$	Standard Error	Comparison group			
			DOM	AR	SL	ISO
DOM	0.3930	0.0041	–	-0.0868 (*p* = 0.3116)	-0.1698 (*p* = 0.0207)	-0.1001 (*p* = 0.1363)
AR	0.4798	0.0039		–	-0.0830 (*p* = 0.2135	-0.0132 (*p* = 0.8284)
SL	0.5628	0.0022			–	0.0697 (*p* = 0.0748)
ISO	0.4931	0.0011				–
$\hat{R}=1.6639$						

Another example was a recent study from a cross-sectional, population-based sample in Iran assessing the prevalence of avoidable blindness [11]. Nearly 3000 persons were examined and blindness was assessed for seven age groups (Table 7). Test statistics $T_{LR}^{2}=134.7$, $T_{W}^{2}=89.1$, $T_{SC}^{2}=161.1$, and *T* = 202.0 consistently show the significant age differences (p-values < 0.0001), and MLE $\hat{R}=3.35$ (${\hat{\rho }}_{i}=\frac{{\hat{\pi }}_{i}}{1-{\hat{\pi }}_{i}}(\hat{R}-1)$ are from 0.05 to 0.59) shows positive correlations between eyes for the same person.

**Table 7:** Prevalence of avoidable blindness from a sample population in Iran [11].

Age Group	Blindness			Sample Prevalence	MLE
	None	Unilateral	Bilaterial		
50–54 yrs	964	23	2	0.014	0.014
55–59 yrs	541	17	8	0.029	0.030
60–64 yrs	469	18	4	0.026	0.027
65–69 yrs	257	16	5	0.047	0.048
70–74 yrs	242	32	3	0.069	0.067
75–79 yrs	127	30	9	0.145	0.134
80+ yrs	104	29	10	0.171	0.149

## Concluding remarks

In this article, we investigated three procedures for testing the homogeneity of correlated data with a cluster size of two. We derived the maximum likelihood estimate algorithm by utilizing the root of third order polynomial equations. The Fisher scoring method is usually criticized for converging slowly, especially when the number of parameters is large (e.g. *g* is large). However, the algorithm derived in this paper is very efficient. This is because only *R* is updated by Fisher scoring iterations, and *π*_*i*_, *i* = 1, …, *g* are the roots of third order polynomials, a closed form solution.

Simulation results showed that the proposed approach (score test) has satisfactory type I error control and reasonable power, regardless of number of groups, sample size, or parameter configurations. On the other hand, the LR test and Wald test have inflated type I error in small sample size. When sample size becomes larger, the three test procedures get closer.

For binary correlated data, there are alternative ways to solve the MLE iteratively or perform hypothesis testing by model-based methods, e.g., GENMOD or GLIMMIX in SAS. However, neither iterative version of test statistics nor model-based method provides the explicit form of the test statistic. The explicit form of the test statistic in our proposed method is useful not only for its simplicity, but also for further development of the exact test. For example, in small sample situation, an exact test may overcome the inflated type I error rate and thus is highly desirable. To perform exact test, it will requires extensive calculations which makes it nearly impossible using iterative versions of test statistics or model-based methods.

To overcome inflated type I error control in asymptotic tests, [10] and [12] considered exact tests for *g* = 2. We consider the exact tests for *g* > 2 as interesting future work.

A user-friendly web-based calculator, including model estimations, hypothesis testings, and simulations, is available from the corresponding author upon request.
